# Gut-Brain Neuroendocrine Signaling Under Conditions of Stress—Focus on Food Intake-Regulatory Mediators

**DOI:** 10.3389/fendo.2018.00498

**Published:** 2018-08-28

**Authors:** Andreas Stengel, Yvette Taché

**Affiliations:** ^1^Department of Psychosomatic Medicine and Psychotherapy, University Hospital Tübingen, Tübingen, Germany; ^2^Charité Center for Internal Medicine and Dermatology, Department for Psychosomatic Medicine, Charité - Universitätsmedizin Berlin, Corporate Member of Freie Universität Berlin, Humboldt-Universität zu Berlin and Berlin Institute of Health, Berlin, Germany; ^3^CURE/Digestive Diseases Research Center, Vatche and Tamar Manoukian Digestive Diseases Division, David Geffen School of Medicine, University of California, Los Angeles, Los Angeles, CA, United States; ^4^VA Greater Los Angeles Health Care System, Los Angeles, CA, United States

**Keywords:** food intake, gastrointestinal functions, gut-brain axis, hypothalamus, peptides

## Abstract

The gut-brain axis represents a bidirectional communication route between the gut and the central nervous system comprised of neuronal as well as humoral signaling. This system plays an important role in the regulation of gastrointestinal as well as homeostatic functions such as hunger and satiety. Recent years also witnessed an increased knowledge on the modulation of this axis under conditions of exogenous or endogenous stressors. The present review will discuss the alterations of neuroendocrine gut-brain signaling under conditions of stress and the respective implications for the regulation of food intake.

## Introduction

Peripheral signals reach the brain *via* neuronal and humoral pathways. The neuronal connection from the gut to the brain through vagal afferents originating from pseudo-unipolar cell bodies located in the nodose ganglia is the most extensively investigated (1, 2). The vagus nerve is composed of over 80% of afferent fibers which convey chemical and mechanosensory signals involved in the regulation of food intake and body weight (3). Peptide hormones predominantly produced in the gut interact with cognate G protein seven transmembrane domain receptors localized on nodose ganglia neurons (4). The expression of these receptors is modulated by feeding and fasting (2, 5) underlining the importance of vagal pathways in the control of energy homeostasis.

Stress influences the expression or circulating levels of several gastrointestinal peptides involved in the regulation of metabolic status under conditions of hunger or satiety (6). The impact of these alterations on the stress response has subsequently been investigated. The present review will highlight the impact of stress on peptidergic gut-brain hormones primarily involved in the regulation of food intake along with the functional implications.

## Modulation of gut-brain signaling under conditions of stress

### Ghrelin

Ghrelin has been identified in the rat stomach (7) which is by far the major site of synthesis as indicated by the pronounced decrease of circulating ghrelin levels following gastrectomy (8). Ghrelin is produced in gastric endocrine X/A-like cells (human nomenclature: P/D_1_ cells) (9) and bears a unique fatty acid residue on its third amino acid essential to bind to its receptor, the growth hormone secretagogue receptor 1a (GHSR1a) (7) now also designated as the ghrelin receptor (GRLN) (10). The enzyme catalyzing this acylation was identified later on and named ghrelin-*O*-acyltransferase (GOAT) (11, 12). Double labeling showed that GOAT immunoreactive cells co-labeled with ghrelin expression in rodents (13) and humans (14). In addition, the finding that GOAT was detected in the pancreas (15) and circulation of rodents (13) and humans (16) supports additional extragastric acylation of the peptide.

Early on, ghrelin has been reported to stimulate food intake after peripheral and central injection in animals (17) and peripheral infusion in humans (18) leading to an increased body weight after repeated injections (19). Expression of the GHSR1a on vagal afferents and the blunting of the peptide's orexigenic action by vagotomy or selective reduction of the GHSR1a in nodose ganglia support a major role of the vagus in mediating ghrelin's action in rats (20, 21). Nonetheless, ghrelin was also shown to cross the blood-brain barrier in both directions (22) indicative of an additional humoral mode of signaling. In the hypothalamus, the GHSR1a is expressed on neuropeptide Y (NPY)/agouti-related peptide (AgRP) neurons of the arcuate nucleus (23, 24). Neuroanatomical and functional studies using optogenetics indicate that ghrelin expressed in axon terminals innervating hypothalamic nuclei increases NPY/AgRP activity (25, 26). In addition, the ghrelin-induced stimulation of food intake is abolished in NPY/AgRP knockout mice (27) demonstrating an essential role of these signaling pathways in mediating ghrelin's orexigenic action in the hypothalamus.

The predominant form of circulating ghrelin is, however, the non-acylated form, des-acyl ghrelin (28, 29). Des-acyl ghrelin initially received little attention due to its lack of affinity to the GHSR1a (7). Nonetheless, des-acyl ghrelin exhibits several biological actions such as decreasing anxiety after intraperitoneral injection in receptor knockout mice (30). Peripheral or intracerebroventricular pretreatment with des-acyl ghrelin blunts the orexigenic action of ghrelin in rats (31) and mice (32). Des-acyl ghrelin's action takes place in a subset of arcuate nucleus neurons distinct from those activated by ghrelin (32). Studies using fluorescein des-acyl ghrelin injected intracerebroventricularly in mice demonstrate that the peptide binds selectively and mainly on arcuate neurons in a GHSR1a independent manner (32). However, to date the receptor mediating des-acyl ghrelin's effects remains to be identified.

Exposure to various acute or chronic stressors influences ghrelin expression and circulating levels although the response varies with the modality of stressors and experimental conditions as detailed in a previous review (6). Tail pinch or starvation increases gastric ghrelin mRNA expression in mice (33). Several other acute stressors including psychological (water avoidance stress, trier social stress test), physical (cold ambient temperature, restraint at 18° C, cholecystectomy, colectomy, cold pressure test) or metabolic (fasting) increase circulating ghrelin levels (34–43). It is to note that in the clinical setting the acute social stress test-induced rise in circulating ghrelin and cortisol levels was not associated with binge eating (35). Likewise, chronic stressors such as repeated restraint in rats, social defeat in mice or trauma in humans also induce a sustained elevation of circulating ghrelin levels lasting for months after the cessation of the stress making ghrelin a persistent biomarker of chronic stress (44–50). The rise in ghrelin may represent a compensatory action to counteract chronic stress-induced anxiety and depression-like behavior (49, 51, 52). Indeed, ghrelin increased the rewarding aspect of food (46) and body weight observed under these conditions, effects no longer observed in GHSR1a knockout mice (47).

By contrast, conditions of stress associated with inflammation decrease ghrelin expression or circulating levels (6). In detail, immune stress triggered by intraperitoneal injection of lipopolysaccharide results in a rapid decline in ghrelin levels associated with a decrease in circulating GOAT protein concentration likely contributing to the reduced acylation in rats (53). Abdominal surgery associated with gastric inflammation (54) decreases acyl and des-acyl ghrelin levels (55, 56) and food intake (57) in rats, an effect blunted by rikkunshito, a herbal medicine stimulating the release of ghrelin (58). Central vagal stimulation, which normalizes the gastric inflammatory response (54), prevents the reduction of plasma ghrelin (55). Chronic inflammatory stress elicited by adjuvant-induced arthritis in rats or rheumatoid arthritis in humans reduces circulating ghrelin levels (59). There are also reports that a psychological stressor such as novelty stress in mice decreases plasma levels of ghrelin and food intake, alterations prevented by rikkunshito (60, 61). Chronic restraint stress or exposure to foot-shock downregulates ghrelin mRNA expression in the mouse hypothalamus (62) and reduces plasma levels of ghrelin in rats (63). These alterations were associated with decreased food intake and body weight gain in mice (62).

Whether the differential alterations of ghrelin by stressors reflect differences in species, metabolic status and/or stressor-related specific recruitment of central and/or peripheral signaling pathways regulating ghrelin release (56, 64) warrant further investigations. Moreover, it cannot be ruled out that difference in modalities to determine ghrelin (total vs. acyl, radioimmunoassay vs. enzyme-linked immunosorbent assay, commercial vs. custom-made kits) might also affect the levels reported.

However, while stressors modulate circulating ghrelin levels, there is also evidence that ghrelin stimulates the hypothalamic-pituitary adrenal axis (33). The peptide injected peripherally upregulates hypothalamic corticotropin-releasing factor (CRF) (33), a key peptide involved in the stress response (65). Recent studies indicate that ghrelin acts *via* the inhibition of hypothalamic GABAergic signaling on CRF neurons in the paraventricular nucleus of the hypothalamus (PVN) (66). In hypothalamic 4B cells *in vitro*, ghrelin stimulates CRF promoter activity through activation of protein kinase A and phospholipase C pathways resulting in increased CRF mRNA levels (67). These data suggest a bidirectional interaction between CRF and the ghrelin signaling system.

### Nesfatin-1

Nesfatin-1 has been first detected in the rat hypothalamus as an 82-amino acid peptide derived from nucleobindin2 (NUCB2) (68). Subsequent research showed a more widespread brain distribution (69) as well as a 10-fold higher expression of NUCB2 mRNA in the stomach indicating that the upper gut is a major site of production (70). Interestingly, immunohistochemical double labeling showed that NUCB2/nesfatin-1 (the antibody recognizes both nesfatin-1 and the full length NUCB2) co-localizes with ghrelin indicating the production in the same gastric endocrine cell type, namely X/A-like cells in rats (70). This finding was later confirmed in humans where these cells are named P/D_1_ cells (14). Nesfatin-1 is able to reach the brain humorally but likely also acts *via* the vagus nerve as intraperitoneal injection induces Fos expression in neurons of the nucleus of the solitary tract that receives input from vagal afferents (71). However, the putative receptor mediating nesfatin-1's effects is still unknown. Converging evidence points toward a G protein-coupled receptor (72, 73). A recent autoradiographic study indicates widespread binding of ^125^I-labeled nesfatin-1 in the brain with signals in the cortex, PVN, area postrema, dorsal motor nucleus of the vagus nerve and cerebellum (74) supporting its centrally mediated pleiotropic effects (75).

The anorexigenic effect of nesfatin-1 has been early on described in several species including rats (68, 76), mice (77), chicks (78), and goldfish (79) following intracerebroventricular injection. In contrast to the convergent findings on the robust food intake-reducing effects of centrally injected nesfatin-1, only one study in mice reported an anorexigenic effect after acute intraperitoneal injection of nesfatin-1 at high doses (71), while other studies in rats (76) and mice (77) showed no effect. Similarly, data following chronic peripheral administration did not produce consistent results: while a reduction of food intake was observed in rats (80), no effect was detected in mice (81). Taken together, the effect of peripheral nesfatin-1 on food intake seems less robust and may not be the primary function of peripherally produced nesfatin-1. By contrast, consistent reports showed that the peptide may play an important role in glucose-stimulated pancreatic insulin release in rats (82) and humans (83).

Convergent findings support an involvement of nesfatin-1 in the stress response. First, several stressors activate nesfatin-1 immunoreactive neurons in the brain, namely psychological (restraint, water avoidance stress) (84–87), physical (abdominal surgery) (88), immunological (injection of lipopolysaccharide) (89) as well as a combination of stressors (chronic variable mild stress) (90). Second, water avoidance stress (91) and injection of lipopolysaccharide (92) elevate circulating levels of nesfatin-1 likely due to the release of the peptide associated with the upregulation of NUCB2 mRNA expression assessed by RT-qPCR and NUCB2/nesfatin-1 protein concentration measured by Western blot in the stomach (92). Third, intracerebroventricular injection of nesfatin-1 increases plasma adrenocorticotropic hormone (ACTH) and corticosterone in rats, an effect likely occurring in the hypothalamus as *in vitro* nesfatin-1 stimulates cytosolic Ca^2+^ in CRF-containing cells of the PVN (86). Therefore, nesfatin-1 exerts its stress-mediating effect likely *via* downstream CRF signaling. Lastly, circulating NUCB2/nesfatin-1 levels are positively correlated with perceived stress in a human female obese population (93) suggesting a potential role in the mediation of stress in humans as well. Interestingly, suicide victims showed altered NUCB2 mRNA expression in a midbrain nucleus implicated in stress-related mood alterations, the Edinger-Westphal nucleus, with an 1.8-fold increase in males and a 2.7-fold decrease in females compared to control subjects who died without any diagnosed neurodegenerative or psychiatric disorder (94).

### Urocortins

Belonging to the CRF family, urocortins (Ucns) have been identified, namely Ucn1, a 40-amino acid (aa) peptide sharing 45% sequence identity with rat/human (r/h) CRF (95), Ucn2, a 39-aa peptide sharing 34% identity with r/h CRF and 42% with r/h Ucn1 (96, 97) and Ucn3, a 38-aa peptide sharing 26% homology with r/h CRF and 21% with r/h Ucn1 (98). Ucn1 binds to both CRF receptors, CRF_1_ and CRF_2_, with equal affinity, whereas Ucn2 and Ucn3 bind to the CRF_2_ receptor with high selectivity (99).

Besides their widespread brain distribution extensively reviewed elsewhere (100), Ucns are also expressed in the periphery, namely the heart, skeletal muscle, spleen, kidney, adipose tissue, ovary, skin (101) as well as the gastrointestinal tract including liver, pancreas, stomach, small, and large intestine (101–108).

Peripheral injection of Ucn1 inhibits food intake in different species including mice (109–111) and sheep (112). In rodents, Ucn1 reduces meal frequency and size and can induce conditioned taste aversion (113) and reduces body weight upon repeated peripheral administration (109). Reports showed that the food intake-reducing effect of Ucn1 is more potent compared to that of CRF, Ucn2, Ucn3, cholecystokinin (CCK) and leptin (109, 110, 113); moreover, a synergistic interaction between Ucn1 and CCK on satiety has been demonstrated (114). The anorexia induced by peripheral Ucn1 is mediated *via* the CRF_2_ receptor based on the observation that the selective CRF_2_ antagonists, antisauvagine-30 and astressin_2_-B, unlike selective CRF_1_ antagonists, suppress the Ucn1-induced reduction of food intake (110, 112, 115). The finding that CRF_2_ knockout mice do not display a reduction of food intake after intraperitoneal injection of Ucn1 further corroborates the implication of this receptor subtype (115). The mechanism through which peripherally injected Ucn1 influences food intake is still to be elucidated. It is unlikely to be mediated by vagal afferent signaling as capsaicin treatment did not alter the anorexigenic response of the peptide in mice (110). Moreover, Ucn1 barely enters the brain through the blood-brain barrier (116). However, CRF_2_ receptors are densely expressed in brain areas outside of the blood-brain barrier, namely the area postrema (117, 118), and neurons at this site are activated by peripheral Ucn1 (119) suggesting a possible pathway.

Ucn2 and Ucn3 injected peripherally also reduce *ad libitum* food intake during the dark phase as well as the refeeding response to a fast with Ucn2 being more potent compared to Ucn3 in mice (110, 111, 114, 115, 120), rats (113), and fish (121). The automated dark phase food intake monitoring showed that Ucn2 reduces meal size (increased satiation), while meal frequency (indicative of satiety) is not altered in mice (115). Interestingly, under re-feeding conditions after a fast, meal size is also reduced, however, meal frequency is increased (decreased satiety) (115). It is important to note that Ucn2, unlike Unc1, does not induce signs of taste aversion (113) pointing toward a specific food intake-reducing effect. Moreover, Ucn2 acts synergistically with CCK to reduce food intake, an effect also observed *in vitro* when recording gastric vagal afferent activity (114). This supports a vagal mode of transmission corroborated by the expression of the CRF_2_ receptor in the nodose ganglia (122, 123).

Various stressors upregulate the peripheral expression of Ucns. Injection of lipopolysaccharide, an immunological stressor, increases the expression of Ucn1, Ucn2 and Ucn3 mRNA in gastric mucosa and submucosa plus muscle layers (107) which is associated with the reduction of food intake under these conditions (53). Ucn1 and Ucn3 immunoreactivity in blood vessels and submucous neurons of the ileum is also increased following *Schistosoma mansoni*-induced inflammation (124). Likewise, blood monocyte-derived dendritic cells display largely increased Ucn1 mRNA and protein expression following stimulation with *Bacteroides vulgatus* or *Fusobacterium varium* (125). There is also evidence that psychological stressors (chronic social stress) upregulates Ucn2 mRNA expression in the pig colon (126), and maternal deprivation increases duodenal Ucn2 and CRF_2_ receptor mRNA, whereas CRF_1_ mRNA is decreased in rats (127).

### Cholecystokinin

CCK is mainly produced in I cells scattered within the upper small intestine with more prominent distribution in the duodenum (128). These cells harbor the feature to be in direct contact with other cells *via* pseudopods (129). Several forms of CCK have been detected including CCK-5,−7,−8,−18,−22,−25,−33,−39, and−58 (representing the number of amino acids) with CCK-8 being the most commonly studied form (128). The demonstration that CCK-58 is the only form detected in the circulation when using a new method for blood processing suggests that the shorter forms are products of degradation (29).

The first described biological action of CCK was the stimulation of gallbladder contraction along with the stimulation of the production and release of pancreatic enzymes and secretion [for review see Sayegh (128)]. The food intake-suppressing effect of CCK was initially reported in rats, and later extended to rabbits, monkeys, pigs, sheep and humans [for review see Sayegh (128)]. Both forms of CCK, CCK-8 and CCK-58 were shown to decrease dark phase food intake following intraperitoneal injection in *ad libitum* fed rats by reducing meal size (130). However, CCK-58 does not shorten the subsequent inter-meal interval as observed following injection of CCK-8 providing evidence for a more sustained effect of CCK-58 (130). Moreover, CCK also suppresses gastric emptying in rats (131) and humans (132) contributing to its anorexigenic effect.

CCK interacts with two receptor subtypes, CCK_A_ (alimentary), expressed in the gastrointestinal tract and on vagal afferents and CCK_B_ (brain), predominantly expressed in the brain (133). CCK is postprandially released from duodenal I cells with lipids and proteins being the most potent stimulators (134–136). Released CCK binds to vagal CCK_A_ expressing afferents and activates neurons in the nucleus of the solitary tract to inhibit food intake, with vagotomy abolishing both the CCK-induced neuronal activation in the brain (137) as well as the anorexigenic effect (138).

A combination of immunological stress using infection with *Giardia lamblia* and psychological stress using the water avoidance model increases CCK levels in the colonic mucosa of mice (139). The stress-induced visceral hypersensitivity could be blocked using the CCK_A_ antagonist, L-364718, and the CCK_B_ antagonist, L-365260 following psychological but not immunological stress (139) giving rise to a role of CCK in visceral sensitivity under selective stress conditions. By contrast, acute or chronic intraperitoneal injection of CCK exerts a protective effect on the impairment of memory functions under conditions of chronic restraint stress (140, 141). Moreover, OLETF rat pups lacking the CCK_A_ display a higher separation-induced ultrasonic vocalization (142) as a surrogate for increased experience of stress. The link between the stress response and CCK signaling was further corroborated by the observation that a well-established immunological stressor, lipopolysaccharide, increases CCK mRNA expression in PVN CRF-containing neurons (143). Intraperitoneal injection of CCK stimulates neuronal activation in noradrenergic A2 neurons (144) as well as increases corticosterone levels to comparable magnitudes observed after injection of CRF (145). Also repetitive intracerebroventricular injections of cortagine, a CRF_1_ agonist, increases CCK mRNA as well as CCK_B_ protein expression in the mouse amygdala and hippocampus resulting in heightened anxiety behavior as assessed using the elevated plus maze and open field test, an effect reversed by intracerebroventricular injection of the CCK_B_ antagonist, LY225910 (146). This anxiety-inducing effect of CCK has also been observed in pharmacological provocation studies following intracerebroventricular injection of CCK-8 in rats that reduced exploratory behavior in the light/dark paradigm (147). In humans, intravenous injection of CCK-4 was shown to induce anxiety and panic symptoms (148, 149). Lastly, tail pinch stress-induced eating in rats (150) is reduced by intracerebroventricular injection of CCK-8 (151). Collectively these observations are indicative of a modulation of the stress response by CCK signaling.

### Glucagon-like peptide 1

Glucagon-like peptide (GLP-1) is produced by endocrine L cells of the small intestine and processed into two biologically active forms, GLP-1_7−36_ amide and GLP-1_7−37_ (152) with GLP-1_7−36_ amide being the predominant form in the human circulation (153). GLP-1 is released postprandially with a biphasic pattern: an early peak of GLP-1 secretion occurs ~15 min after meal intake that involves humoral (154, 155) and vagal (156) stimulation, while a later and larger peak is related to the direct contact of L cells with food components (157).

Peripheral but also central administration of GLP-1, in addition to the well-described incretin effect, results in a decrease of food intake in animals (157–159) and humans (160). In addition, the slowing of gastric and intestinal transit (161, 162) is likely to contribute to the food intake-reducing effect.

GLP-1 signals to the brain *via* the vagus nerve expressing the GLP-1 receptor (163) as shown by the suppression of the anorexigenic effect of peripherally injected GLP-1 by vagotomy (164, 165). It is to note that GLP-1 is also expressed in the brainstem nucleus of the solitary tract that projects to the PVN (166), and local knockdown of the pro-glucagon gene in the nucleus of the solitary tract increases food intake and also body weight gain (167). Since lesioning of these connections blunts the anorexigenic effect of peripherally injected GLP-1 (164) the gut-vagal-brainstem-hypothalamus connection is essential for the mediation of GLP-1's food intake-suppressing effect. Nonetheless, GLP-1 is able to cross the blood-brain barrier by simple diffusion (168). However, the rapid degradation of the peptide by circulating DPPIV (169) points toward the importance of neural and/or paracrine signaling.

GLP-1 can modulate a number of stress responses. Under basal conditions, GLP-1_7−36_ amide injected peripherally stimulates circulating corticosterone levels in mice and rats as well as cortisol levels in healthy human subjects (170). Other studies showed that targeted knockdown of the GLP-1 receptor in single-minded 1-expressing neurons of the PVN reduces hypothalamic-pituitary-adrenal axis responses to acute and chronic stress and this was associated with reduced anxiety-like behavior and a prevention of body weight reduction under conditions of chronic stress (171). Similarly, injection of the GLP-1 receptor inverse agonist, exendin-_(9−39)_ into the dorsal subregion of the lateral septum blocks the acute restraint stress-induced anorexigenic effect in rats (172). While these studies support GLP-1's permissive role in the activation of stress signaling pathways, other reports showed that mice lacking the GLP-1 receptor display an increased corticosterone response to novel environment stress (173).

Several protective effects of GLP-1 have been reported under conditions of stress. GLP-1 injected intracerebroventricularly prevents gastric mucosal lesions induced by a combination of cold and restraint stress, an effect blocked by exendin-_(9−39)_ (174). Subcutaneous injection of liraglutide, a GLP-1 analog, inhibits visceral allodynia induced by injection of lipopolysaccharide or repeated water avoidance stress (175). In humans with alcohol dependence, treatment with the GABA-B receptor agonist, baclofen at a dose of 30 mg/day increases circulating levels of GLP-1 (176), possibly associated with a reduced craving for alcohol. Moreover, GLP-1 receptor activation reverses the restraint stress-induced activation of bone marrow sca-1^high^c-Kit^high^CD48^low^CD150^high^ proliferation of hematopoietic stem cells in mice, thereby reducing the inflammatory response (177). In a study using geniposide as GLP-1 agonist these anti-inflammatory effects were associated with an amelioration of depression-like behaviors following repeated restraint stress (178). Also *in vitro* GLP-1_7−36_ prevents various stressors (e.g., H_2_O_2_ and amyloid β_1−42_)-induced death of murine hippocampal HT22 cells, an effect likely mediated *via* increased phosphorylation of Akt and ERK1/2 (179).

### Peptide YY

Peptide YY (PYY) is derived from L cells located in the distal small intestine and colon (180). The peptide circulates in two forms, PYY_1−36_ and PYY_3−36_, which is the predominant form in the blood (181) resulting from the processing by dipeptidyl peptidase IV (182). PYY is well established to reduce food intake in animals and humans following peripheral injection *via* binding to the Y_2_ receptor (183). This was demonstrated by the blunting of the peptide's anorexigenic effect by the Y_2_ antagonist, BIIE0246 (184) and knockout of the Y_2_ receptor (183) in rodents. The anorexigenic gut-brain mode of action may involve the vagus nerve and humoral pathways. The Y_2_ receptor is expressed on vagal afferents (185) and vagotomy blocks the anorexigenic effect of PYY (186) In addition, PYY can also cross the blood-brain barrier in a non-saturable manner (187). There is evidence that peripherally injected PYY or PYY_3−36_ activates brain nuclei such as the nucleus of the solitary tract (188) and hypothalamic nuclei (189) which are known to regulate food intake. In the brain, PYY microinjected directly into the arcuate nucleus, a nucleus involved in the regulation of food intake and expressing the Y_2_ receptor (190), reduces food intake. This is achieved by decreasing the activity of neuropeptide Y-containing neurons and activating proopiomelanocortin-containing cells (183).

Repetitive water avoidance stress decreases circulating PYY levels compared to non-stressed rats (191). Likewise, in humans a well-established psychological stressor, the Trier social stress test, reduces circulating PYY levels in normal weight and obese women (192). However, in mice, water immersion stress results in increased plasma PYY levels (193). Other studies showed that mice lacking PYY have an enhanced restraint stress-induced fecal pellet output and upper gastrointestinal transit (194); therefore, the peptide might play a modulatory role in the stress response. Whether the contrasting effects of stress on PYY release are related to species or stress modality differences remains to be further investigated.

## Summary

**Figure 1:**
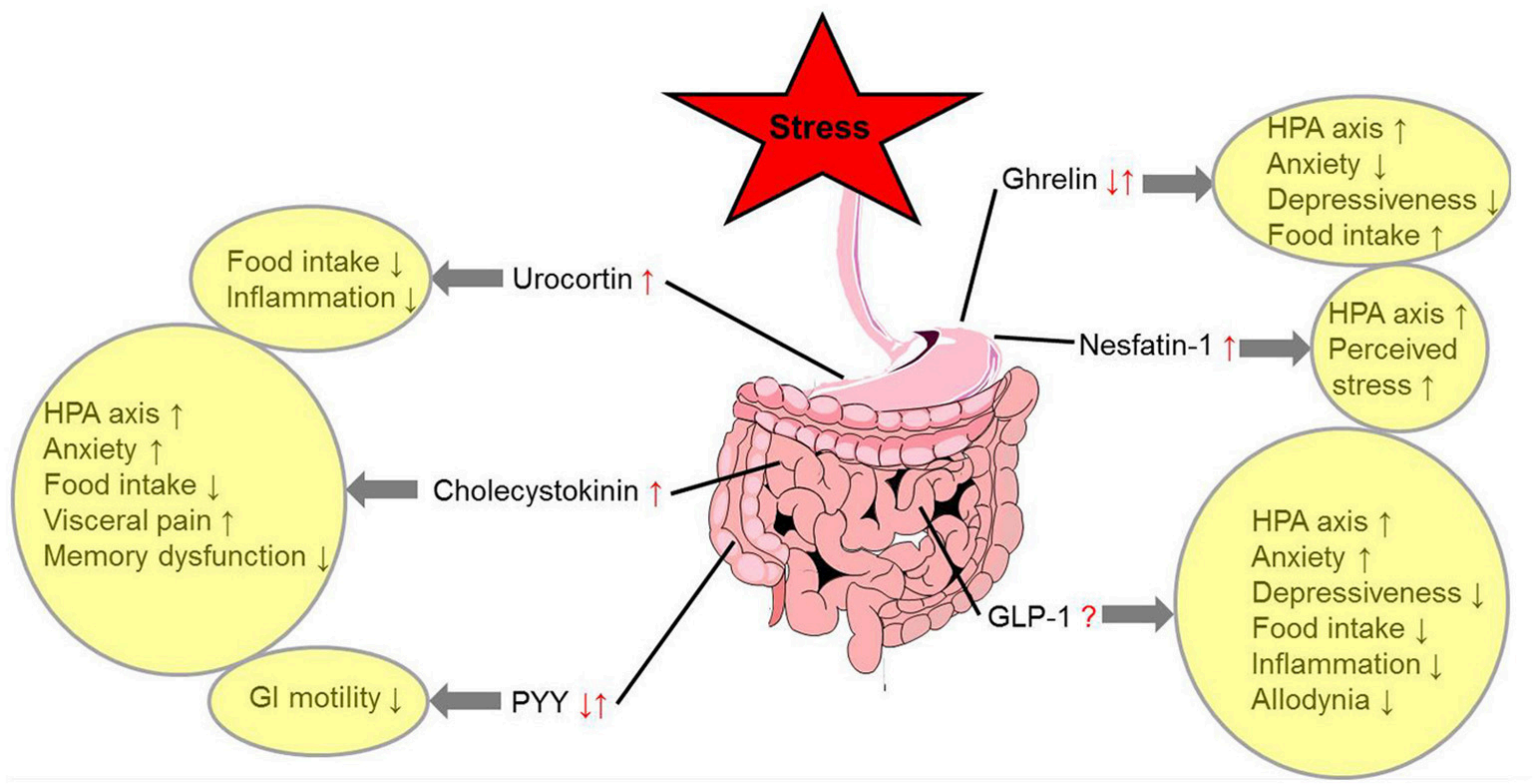
Alterations of gut-brain peptides under conditions of stress and functional implications. ↓, decrease; ↑, increase; ?, unknown effect; GLP-1, glucagon-like peptide 1; GI, gastrointestinal; HPA axis, hypothalamus-pituitary-adrenal axis; PYY, peptide YY.

Various stressors alter the expression or circulating levels of several gut-brain peptidergic hormones involved in the regulation of hunger and satiety. While most anorexigenic peptides are upregulated under conditions of stress (nesfatin-1, Ucns, and CCK), others were shown to be differentially regulated dependent on the type of stressors (ghrelin and PYY), and for GLP-1 conclusive data are lacking so far. In addition, there is a further activation of the hypothalamus-pituitary-adrenal axis induced by specific gut peptides (ghrelin, nesfatin-1, CCK, and GLP-1) acting *via* neuronal and/or humoral gut-brain signaling highlighting the PVN as key responsive area orchestrating the stress response. This results in an increased perception of stress (nesfatin-1) and an alteration of anxiety and depressiveness (ghrelin, CCK, and GLP-1) with the PVN and Edinger-Westphal nucleus playing an important role in the behavioral responses. While most peptides contribute to stress-induced anorexia (Ucns, CCK, and GLP-1), ghrelin can stimulate food intake under these conditions (Figure 1). Despite the fact that our knowledge on these regulatory pathways greatly increased during the past years, the interactions between these peptides (114, 195) under stress conditions should be further investigated along with the possible translation of these findings—derived mainly from animal models—to humans.

## Author contributions

AS drafted the manuscript. AS and YT reviewed and finalized the manuscript.

### Conflict of interest statement

The authors declare that the research was conducted in the absence of any commercial or financial relationships that could be construed as a potential conflict of interest.
